# Profiling Transcripts of Vector Competence between Two Different *Aedes aegypti* Populations in Florida

**DOI:** 10.3390/v12080823

**Published:** 2020-07-29

**Authors:** Dongyoung Shin, Seokyoung Kang, Chelsea T. Smartt

**Affiliations:** 1 Florida Medical Entomology Laboratory, Department of Entomology and Nematology, Institute of Food and Agricultural Science, University of Florida, Vero Beach, FL 32962, USA; 2 Department of Infectious Disease and Immunology, College of Veterinary Medicine, University of Florida, Gainesville, FL 32611, USA; skang1@ufl.edu

**Keywords:** chikungunya virus 1, *Aedes aegypti* 2, wnt signaling pathway 3, notch signaling pathway 4

## Abstract

A Chikungunya virus (CHIKV) outbreak in Italy in 2007 spread to include the islands of the Caribbean and most of the Americas and still circulates in Europe and Africa. Florida being close in distance to the Caribbean islands experienced a CHIKV outbreak in 2014 and continues to have a few travel-related cases each year. It is known that different environmental conditions in different regions can result in genetic variation that favor changes in competence to arbovirus. We evaluated the vector competence of Florida *Aedes aegypti* for CHIKV and determined if there is a geographic component that influences genes involved in CHIKV competence. We utilized a genomic approach to identify the candidate genes using RNA sequencing. The infection and dissemination results showed that field populations were more competent vectors for CHIKV than a lab population. The differentially expressed genes in the two field-collected CHIKV-infected populations, compared to the Rockefeller strain, were related to the Wnt/Notch signaling pathway, with similarity to genes scattered throughout the signaling pathway. This result suggested the possibility of identifying genes involved in the determination of vector competence in different gene pools of *Ae. aegypti*.

## 1. Introduction

Chikungunya virus (CHIKV) is an emerging mosquito-borne virus discovered in Tanzania in 1952, and the symptoms of the infection in humans are fever and joint pain [1]. CHIKV outbreaks have occurred in the coastal region of Kenya and in the Indian Ocean Islands and India. In addition, it recently spread, following an outbreak in Italy in 2007, to an outbreak in the islands of the Caribbean, Europe and Africa. Later a CHIKV outbreak in St. Martin Island spread to the Americas [2]. Florida experienced a CHIKV outbreak in 2014 and a few travel-related cases of CHIKV infection have been reported since then in each year [3,4]. With the consistent travel-related CHIKV cases in the U.S., including Florida [4], and the presence of the well-established competent mosquito vectors for CHIKV, Florida is well placed for frequent outbreaks. In fact, we have shown that *Ae. aegypti* mosquitoes collected in Florida can effectively vector CHIKV, and that temperature change influenced both infection and dissemination [5,6]. There exists an immediate need to understand CHIKV and its interaction with local competent mosquito vectors.

During the last decade, CHIKV outbreaks were mainly vectored by *Ae. aegypti* [7]. The warmer tropical environments seemed to favor outbreaks driven by competent *Ae. aegypti* [2,8]. Studies on an outbreak that occurred in 2007 revealed that *Aedes* populations along the coastal regions of Kenya were highly susceptible to infection following an unusually warm dry spell [9]. During that same year, a new genetic variant of CHIKV emerged, which resulted in increased vector competence for *Ae. albopictus* mosquitoes. *Ae. albopictus* are adaptable to temperate environments leading to the spread of CHIKV through Europe [8,10]. In fact, studies on the recent *Ae. albopictus*-driven CHIKV outbreak in Europe implicated the involvement of climate alterations on the success of this invasion [8]. These few studies suggest that the CHIKV–mosquito interaction is highly influenced by environmental alterations. It is also known that biotic and abiotic conditions in different regions and sub-regions of the world result in genetic variation that favor changes in competence to arbovirus including West Nile and dengue virus [11,12,13]. These regional differences may be due to the microbiome or climate under which the mosquitoes developed, exposure to different xenobiotics, or due to genetic isolation [14,15].

In this study, we utilized a genomic approach to identify the candidate genes that might be responsible for vector competence in *Ae. aegypti* to CHIKV using RNA sequencing (RNAseq). The expression profiling was evaluated in *Ae. aegypti* populations from different geographic areas of Florida for competence for CHIKV, and pathways related to competence for CHIKV mapped in an effort to interfere with CHIKV transmission.

## 2. Materials and Methods

### 2.1. Mosquitoes

*Ae. aegypti* populations were collected from two geographic areas in Florida—Key West and Vero Beach—working with the appropriate Mosquito Control District. The collected mosquito larvae were reared under standard insectary conditions. Adult mosquitoes (F1–2; 800 mosquitoes each) were maintained at 28 °C (14/10 h light/dark period, 70% relative humidity) [6] and fed 20% sucrose solution ad libitum. Parental females from field collections were blood fed bovine blood in sausage casings [16] for first generation (F1) offspring (egg) production. We used the F2 generation to ensure that there would be enough sample numbers for this study. A lab strain of *Ae. aegypti* (Rockefeller strain) was also included as a control population to juxtapose field populations for vector competence. Rockefeller strain (ROCK), Vero Beach (Vero) and Key West (KW) populations were subjected to CHIKV infection.

### 2.2. Infection of Adult Female Ae. aegypti

Five- to six-day-old female *Ae. aegypti* from the three different populations (300 females/population) were fed defibrinated bovine blood (Hemostat, Dixon, CA, USA) containing CHIKV (6.82 log10 plaque-forming units (pfu)/mL). This sample size has been shown in past experiments to have sufficient statistical power to detect treatment effects [6]. For vector competence experiments, we utilized the CHIKV strain LR2006-OPY1 (GenBank accession # DQ443544; [6,17]). Infectious blood meals were delivered to *Ae. aegypti* using an artificial feeding apparatus (Hemotek, Lancashire, UK) following previously established methods [6,17,18]. The fully engorged specimens were transferred to 16 oz cardboard cages with mesh screening, maintained in incubators at 28 °C, and provided a 20% sucrose solution ad libitum.

### 2.3. Infection and Dissemination Rates in Different Ae. aegypti Populations

Approximately 15 female mosquitoes from the CHIKV-infected females in each population were collected at 4 days post infection (dpi) for RNAseq. The extrinsic incubation for CHIKV is known to take 2–9 days and the CHIKV dissemination rates were determined within 5 days post infection (dpi), based on previous studies showing the temporal progression of infection in *Ae. aegypti* under these conditions [5,19]. Therefore, gene expression profiling at 4 dpi in this study should allow immediate detection of responses in mosquitoes in each population to enable screens for the main signaling pathway targeted by CHIKV. Each biological replicate contained 5 mosquitoes and the total RNA was extracted separately. At 10 dpi, the remaining mosquito samples (~50 mosquitoes/group) were collected individually and immediately frozen for CHIKV detection assays (Table 1). Bodies and legs of each individual were tested separately for CHIKV RNA by qRT-PCR (forward primer: 5-ACC CGG TAA GAG CGA TGA ACT-3; reverse primer: 5-ACG CCG CAT CCG GTA TGT-3) for determining infection and dissemination rate of each population, respectively. All samples were homogenized using a Tissue Lyser (Qiagen, Valencia, CA, USA) and RNA extracted using Trizol Reagent (Invitrogen, Carlsbad, CA, USA) following previously established methods [13,20]. Quantitative RT-PCR was performed using SsoAdvanced SYBR Green Supermix (Bio-Rad, Hercules, CA, USA) on the Bio-Rad CFX96™ Real-Time PCR Detection System and following the included protocols. The standard curve was generated based on 10-fold serial dilutions of CHIKV and quantified by qRT-PCR, as described above. Chikungunya titer was determined from triplicate quantification cycle (Cq) values using Bio-Rad CFX manager software. The raw data was normalized by Log10 transformation and regression analysis used to determine a qRT-PCR-derived titer (Qpfu/mL) [13].

The different physiological responses, resulting in vector competence of each population from different geographic regions for CHIKV infection and dissemination, were analyzed using analysis of variance (ANOVA) and maximum likelihood categorical analyses of contingency tables (JMP pro13). Separate maximum likelihood tests were used for each measure of infection and dissemination rate, in order to more clearly identify viral barriers that may be modified in different populations from different areas of Florida. Titers in body and leg among three populations were analyzed with ANOVA Dunn’s multiple test (Prism 7).

### 2.4. High-Throughput RNA Sequencing

The extracted total RNA (~1ug/ sample) with three biological replications from each treatment group at 4 dpi was sent to the Center for Genome Technology Sequencing Core, John P. Hussman Institute for Human Genomics in University of Miami Miller School of Medicine for library generation and illumina RNA sequencing.

Nine RNAseq libraries were generated from three populations with three biological replicates for RNA sequencing. The RNA samples were qualified with Agilent BioAnalyzer RNA integrity number (RIN) Score >7. Cluster generation took place on the Illumina cBot. Sequencing took place on the Illumina HiSeq2000 using the reagents provided in the Illumina TruSeq PE Cluster Kit v3 and the TruSeq SBS Kit–HS kit. The FASTQs were processed to remove Illumina adaptors using the TrimGalore! software package and the STAR aligner v2.5.0a with default mapping parameters [21]. Aligned reads in SAM format were quantified using the count function of HT-Seq using the AaegL3.3 gene set GTF obtained from VectorBase (www.vectorbase.org). Differential expression between genes was calculated using the EdgeR software package [22]. Normalization for sample-to-sample variability is accounted for by correcting the raw counts for a gene to counts per gene per million bases of sequencing library.

The differentially expressed genes (adjust *p* value < 0.05) were subjected to the GO term Functional analysis using Biomart tool from Vectorbase database (Vectorbase.org), in order to find the enriched function in the differentially expressed genes among three populations. The changed gene expression level can result in either over-expressed or under-expressed genes. The enriched functions in the up-regulated genes and down-regulated genes from the Vero and KW populations were compared to the ROCK population and are represented in Figures 2 and 3.

### 2.5. Validation of Expression Differences between CHIKV in Two Populations of Ae. aegypti

For validation of the RNAseq data, four genes were selected from the differentially expressed gene list (*p* < 0.05). These genes were related to signaling pathway and show different expression levels among populations in this study. The exon sequences of each gene were exported from *Ae. aegypti* complete transcript database (www.vectorbase.org). The primer sets for the four selected genes were designed based on the exon sequences, using IDT PrimerQuest Tool (https://www.idtdna.com/Primerquest/Home/Index) (Table S1). Ribosomal protein gene S7 was used as an endogenous control gene and the selected genes were quantified with qRT-PCR.

## 3. Results

The different *Ae. aegypti* populations presented significantly different proportions of mosquitoes with infected bodies (Chi square = 7.185, degrees of freedom (df) = 2, *p* = 0.0275; Table 1 and Table 2) and disseminated infections (Chi square = 19.262, (df) = 2, *p* < 0.0001; Table 1 and Table 2) on day 10 following a CHIKV infectious blood-meal. The field populations of *Ae. aegypti* from Florida were significantly more likely to be infected than the ROCK population, and infection rates between the two field populations were not significantly different (Table 1 and Table 2). Vero Beach population of *Ae. aegypti* was more likely to have a disseminated infection than either the ROCK or KW population statistically, with no significant difference between ROCK and KW (Table 1 and Table 2). Two field populations had significantly higher body titers at 10 dpi than the ROCK population (*p* < 0.0001; Table 3) but there was no significant difference between body titers of the two field populations. All populations of *Ae. aegypti* had significantly different leg titers for disseminated infection and Vero and KW populations showed higher titers than the ROCK population (*p* < 0.0001; Table 3).

**Figure 1:**
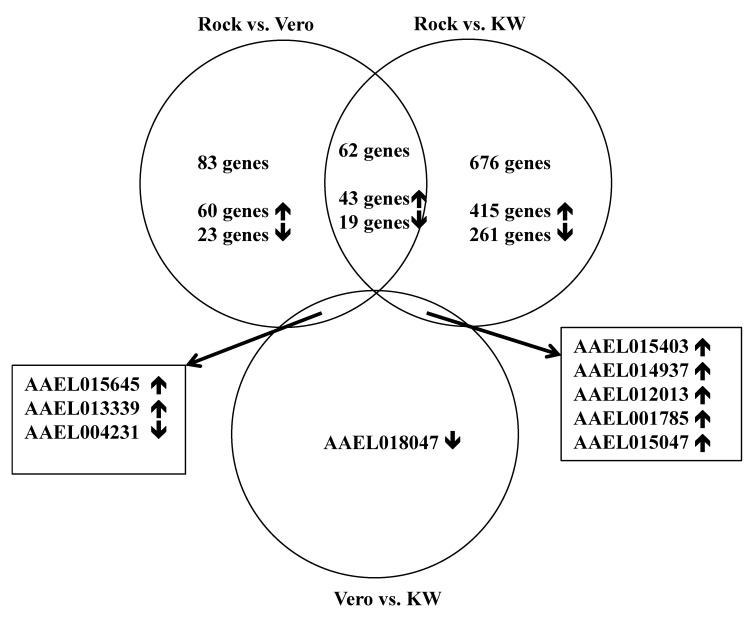
Venn diagram comparing differentially expressed genes among three-paired comparisons from three populations.

**Figure 2:**
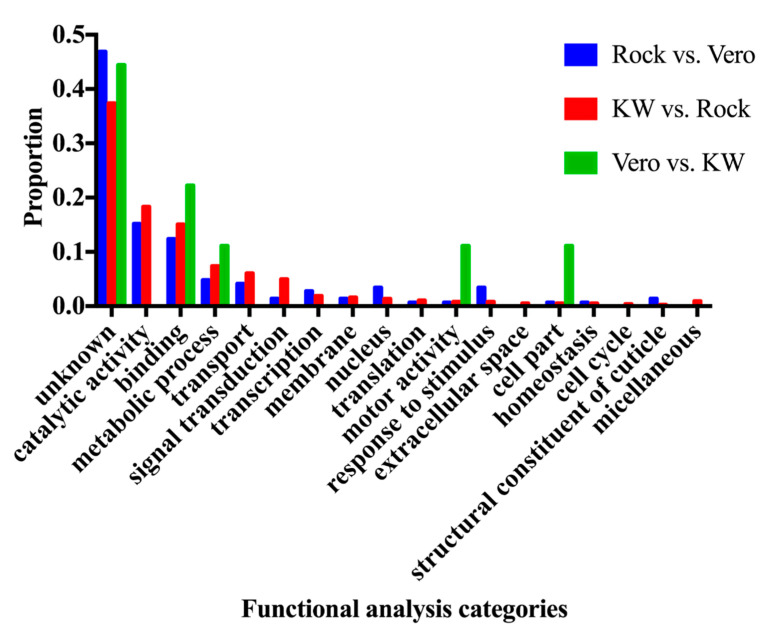
Comparison of three different sets of functional characterizations of significantly differentially expressed transcripts between populations (*p* < 0.05).

**Figure 3:**
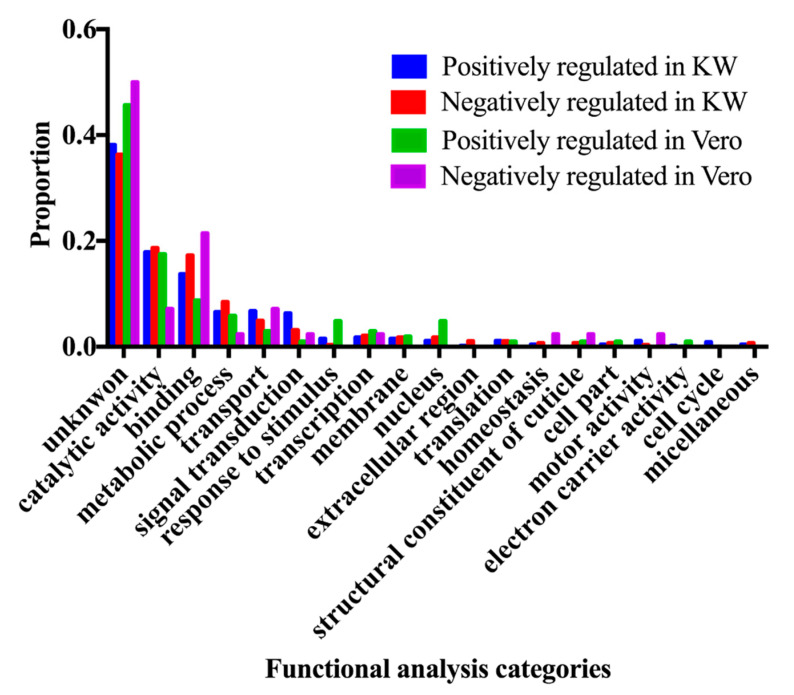
Enriched functional analysis of the regulated expression of the significantly differentially expressed genes (*p* < 0.05).

A total of 216,859,828 reads were generated from triplicate samples from the three populations (Table 4). The obtained reads were archived in GenBank (accession#: SRP136008). We found 145 and 743 differentially expressed genes between the Vero and ROCK and KW and ROCK, respectively (adjusted *p* value <0.05) (Table S2). There were 62 genes with overlapping expression in the two comparison analyses (Vero vs. Rock and KW vs. Rock; Figure 1, Table S3). Two field populations were compared to each other and nine genes were differentially expressed (Table 5). The enriched gene functions were analyzed and compared between each pair of the three populations (Figure 2). Since there were only nine differentially expressed genes between Vero and KW populations, the proportion of enriched functions included in the nine genes were not comparable with the enriched functions in the other pairs used in the analysis. The enriched functions in Vero and KW populations compared to ROCK population were similar in most of the 19 categories searched except for signal transduction, response to stimulus, and structural constituent of cuticle (Figure 2). The up-regulated genes and down-regulated genes of the two field populations were analyzed and compared to the ROCK population (Figure 3). Approximately half of the differentially expressed genes in this study did not have annotated function or annotated as hypothetical genes because the genome project for *Ae. aegypti* has not been completed [23]. The most enriched functions in the differentially expressed genes in Vero and KW populations compared to ROCK population are catalytic activity, transport, and signal transduction (Figure 3, Table 6). Response to stimulus category, which is mainly a general defense function, was a notable difference between the ROCK and Vero populations (Figure 3). The regulation of genes in transport and signal transduction function differs between Vero and KW, where both functions are positively regulated in KW and negatively in Vero (Figure 3, Table 6). Validation of a subset of these genes by qRT-PCR supported the RNAseq results (Table 7), as the expression of all the tested genes corresponded with the RNAseq results (*p* < 0.05).

## 4. Discussion

The infection rates and detection of disseminated infection in mosquito body showed that Vero and KW populations are more competent vectors for CHIKV than ROCK, used widely as a laboratory experimental strain, (Table 1, Table 2 and Table 3), although without transmission rate data overall competence cannot be assessed. The vector competence study results showing different disseminated infection rates of CHIKV support the assumption that vector competence for CHIKV of the Vero and KW population is conferred by differences in the gene pool, because the two populations were collected from geographically different regions [24]. The dissemination rates between the KW and Vero populations were significantly different (<0.05), while both are notably higher than ROCK (Table 1 and Table 2). The infection rate and body titer between the two field populations were not significantly different unlike dissemination rate and leg titer (Table 1, Table 2 and Table 3). Significant differences in disseminated infection rates between field populations suggest any differentially expressed genes discovered by RNAseq analysis could be involved in the dissemination of CHIKV. Although the titer difference in disseminated infection seems small unlike cell culture data (around 10-fold), this can be a biological difference because the results came from RNA from legs pulled from individual mosquitoes and statistically supported with sufficient sample sizes (38–73). Direct comparison analysis between CHIKV infected Vero and KW populations showed expression differences of nine genes and the functional analysis results failed to implicate any specific enriched function known to influence variation in dissemination rate and titer, such as immune response (Table 5). Similarity in infection rates at 4pdi for CHIKV in the Vero and KW populations likely contributed to the smaller number of gene expression differences (Table 1). Although RNAseq data showed only nine genes by comparison analysis between KW and Vero populations, the differentially expressed genes between KW and Vero populations compared to ROCK also represent the variation between KW and Vero populations.

Comparisons between ROCK and other populations with different vector competence to CHIKV should reveal vector competence genes to CHIKV in *Ae. aegypti*. The most enriched gene functions in KW and Vero compared to ROCK are catalytic activity, binding, metabolic process, transport, and signal transduction (Figure 2). These enriched functions in each population can contribute to mosquito vector competence to CHIKV [6]. Catalytic activity and binding categories represent the first and second largest portion in the assigned functions. The catalytic activity group is comprised of enzymes active during blood meal digestion and the binding category includes protein and nucleic acid binding, which is broadly defined as interaction with nucleic acid and protein. These two functions have been commonly shown in studies investigating blood digestion [25], also these functions can be enriched as downstream of the metabolic process cascade. Thus, catalytic activity and the binding categories were likely chosen because of the inclusion of the highly enriched metabolic process category. Metabolic processes were highly enriched in both field populations compared to ROCK. This may happen because the field populations were likely exposed to biotic threats and/or xenobiotics including pesticides, which have inheritable effects on offspring, unlike ROCK, a longstanding lab population. Although this experiment did not test detoxification effects directly, the Vero and KW populations may increase metabolic functions to detoxify or destroy the xenobiotics around the field environment [26], as supported by findings from this study showing many up-regulated cytochrome P450 genes (~2%) in the KW and Vero populations (Table S2). Consequently, field-collected populations may allocate more energy to detoxification and general defense mechanisms for bacterial infection at the expense of either keeping general immune response genes suppressed under the normal condition or induced following pathogen infection [27]. Genes involved in metabolic functions are also known to be involved in mosquito blood digestion [28]. These CHIKV-induced, differentially expressed genes related to detoxification and metabolism can elicit different responses due to the introduction of a pathogen and contribute to vector competence, as has been shown for dengue infection in *Ae. aegypti* mosquitoes [29]. In the midgut tissue of *Ae. aegypti*, differentially regulated genes were involved in several metabolic processes such as protein, lipid and carbohydrate metabolism. Two such genes, transferrin and heat shock protein 60, were reported to play a role in CHIKV survival and replication in the midgut [30].

Response to stimulus category was a notably up-regulated function in the Vero population compared to the KW population (Figure 3). The largest number of genes in the response to stimulus category of the KW population was related to stress from environmental issues. An innate immunity related gene, cecropin, was down regulated in this population. Two different cecropin genes were over expressed and three types of defensin were down regulated within this category in the Vero population. The defensin genes have previously been profiled in this Key West population after CHIKV and DENV infection; thus the defensin family might have an antiviral role in CHIKV infections [31]. Both defensin and cecropin are anti-microbial peptides and they are also involved in innate immunity to DENV and Sindbis virus, suggesting that defensin and cecropin peptides are also related to immunity against CHIKV in *Ae. aegypti* (Table S2).

Signal transduction category showed slightly dissimilar expression in KW and Vero populations, where a notable signaling pathway was present in both, but the proportion of expressed pathway related genes is larger in the KW population. At the same time the KW population showed lower leg titer and low dissemination rates for CHIKV compared to the Vero population (Table 3). This difference in the signal transduction category may be attributed to variability in vector competence between the two field populations. Functional analysis of the RNAseq data revealed the majority of genes in the signaling pathway categories were involved in Notch and Wnt signaling (Table 6). It is well known that the wingless gene is one of the main genes in the Wnt signaling pathway and that the Wnt and Rho signaling pathways closely interact in cell development [32,33,34,35,36]. Based on gene description, functional analysis, and literature review, the differentially expressed genes that relate to Wnt and Notch signaling pathways and identified in the RNAseq analysis in this study are listed in Table 6. Both KW and Vero populations possess differentially expressed Wnt and Notch signaling pathway related genes after CHIKV infection compared to ROCK (Table 6). The Notch signaling pathway has a role in neuronal function and development [37] and silencing the Notch pathway component Delta has been shown to control susceptibility to dengue virus infection in mosquito midguts [38]. In the enriched functions identified in this study, transport and signal transduction included several genes involved in the Wnt pathway including the Rho signaling pathway, which functions in development, motility, and immunity to DENV (Table 7) [34]. Disruption of the Wnt signaling pathway causes many diseases, tumors, and genetic disorders in mammalian systems [34]. Having upregulated Notch signaling pathway expression in *Ae. aegypti* infected with CHIKV in this study suggests involvement in interactions between this mosquito species and CHIKV [38]. In addition to Notch signaling pathway, genes in the Wnt signaling pathway were over-expressed in a Sindbis virus-infected *Drosophila* cell line [39,40]. Additionally, the expression of one of the main components of the Wnt signaling pathway decreased by 41% in *Anopheles gambiae* after infection with O’nyong-nyong virus (ONNV) [41]. The Wnt signaling pathway antagonistically interacts with the Notch signaling pathway for embryonic development in *D. melanogaster* [32].

CHIKV, ONNV, and Sindbis virus belong to the Alphavirus family and Sindbis virus has been used as a model system to study Alphaviruses. Additionally, *Drosophila* has been used as a model system for mosquito research to explore various aspects of mosquito biology, including the immune system, genetics and genomics [42]. A *Drosophila* cell line revealed that the up-regulated Notch signaling pathway was involved in cell growth rate after Sindbis virus infection [40]. In our study, genes involved in the Notch signaling pathway, such as rab GTPase activating protein and neuronal function and development gene, were included in the differentially expressed gene list, showing a similar gene expression pattern with the Sindbis virus model system (Table 6).

Compared to Notch signaling pathway, Wnt signaling pathway related genes were more remarkable in this study. The Rho protein and wingless protein are major regulators of the cytoskeleton in the Wnt signaling pathway and approximately 1.5% of the differentially expressed genes in both Vero and KW populations, directly and indirectly involved in the Wnt pathway, were dissimilar in expression in response to CHIKV infection, supporting Wnt signaling pathway involvement in mosquito vector competence to CHIKV. The Rho GTPase gene in the Wnt pathway was revealed to activate the c-Jun N-terminal kinases (JNK) pathway in vertebrates [43] (Table 6) and the JNK pathway was shown to play a role in immunity in mosquitoes and in mosquito-virus interactions [44,45]. Taken together the results suggest possible co-expression of the JNK signaling pathway with Wnt and Notch signaling pathways as a correlated pathway in vector competence to CHIKV.

The biological difference including metabolism rate and response to various stimuli from the environment can increase mosquito susceptibility to CHIKV. The differentially expressed genes in KW and Vero populations compared to ROCK are related to the Wnt/Notch signaling pathway. Involvement of these pathway genes suggests that Wnt/Notch signaling pathway may play a role in vector competence in Florida populations of *Ae. aegypti* to CHIKV. The Wnt/Notch signaling pathways could be the pathway targeted by alphavirus infections in *Ae. aegypti*. The RNAseq analysis in this study did not include a group fed a non-infectious blood-meal and thus these signaling pathways may also be involved in blood feeding. Further studies including silencing Wnt/Notch signaling pathways along with vector competence studies, with CHIKV and comparison between groups fed infectious and naïve blood meals, will reveal the characteristics of these signaling pathways. Therefore, it is necessary to investigate the mechanism and effector genes in the Wnt/ Notch signaling pathway that interact with CHIKV in *Ae. aegypti*. Additionally, results from this study suggest the possibility of identifying genes involved in determination of vector competence in different gene pools from different populations of *Ae. aegypti.*

## Figures and Tables

**Table 1:** Infection and dissemination rate results for Chikungunya virus infection in three different populations.

Mosquito Population	Infection Rate–4 dpi Body (No. of Tested Mosquito)	Infection Rate–10 dpi Body (No. of Tested Mosquito)	Dissemination Rate–10 dpi Legs (No. of Tested Mosquito)
Rockefeller	73.3% (11/15)	88.4% (38/43)	73.7% (28/38)
Vero Beach	93.3% (14/15)	98.5% (64/65)	100% (64/64)
Key West	86.6% (13/15)	97.3% (73/75)	86.3% (63/73)

**Table 2:** Pearson Chi-square *p*-value on pairs for CHIKV infection and dissemination rate at ten days post-exposure to chikungunya virus.

Population	Infection Rate			Dissemination Rate		
	ROCK	Vero	KW	ROCK	Vero	KW
ROCK	1.0000	0.0250	0.0473	1.0000	<0001	0.0516
Vero	0.0250	1.0000	0.6457	<0001	1.0000	0.0021
KW	0.0473	0.6457	1.0000	0.0516	0.0021	1.0000

**Table 3:** Titer for mosquito body and legs at ten days post-exposure to Chikungunya virus for each mosquito population tested.

Mosquito Population	Mean ± SE log_10_–10 dpi Body	Mean ± SE log_10_–10 dpi Legs
Rockefeller	2.07 ± 0.49 ^a^	2.06 ± 0.30 ^a^
Vero Beach	2.99 ± 1.87 ^b^	3.78 ± 1.07 ^b^
Key West	3.25 ± 1.57 ^b^	3.21 ± 1.33 ^c^

^a,b,c^ Levels not connected by same letter are significantly different.

**Table 4:** Mapping summary.

Population-Replicate	No. of Reads	Average Read Length	Uniquely Mapped Reads	Uniquely Mapped Reads %	Average Mapped Length
ROCK- 1	18323605	235	12570137	68.60%	234
ROCK- 2	38931514	236	25824148	66.33%	236.32
ROCK- 3	21212206	232	15195462	71.64%	233.3
Vero- 1	23852789	234	16833957	70.57%	233.39
Vero- 2	20392873	236	14198158	69.62%	235.17
Vero- 3	21009310	235	14592411	69.46%	233.62
KW- 1	22677415	231	15608399	68.83%	232.27
KW- 2	24292504	231	16551381	68.13%	231.31
KW- 3	26167612	235	16724575	63.91%	233.97
Average	24095536.44	233.89	16455403.11	68.57%	233.71

**Table 5:** Nine genes differentially expressed between two field populations of *Ae. aegypti*.

Transcript ID	Description	Fold-Change (log2)	Function
AAEL015403	Conserved hypothetical protein	6.803267	Protein binding
AAEL015645	Hypothetical protein	4.198290	Nucleic acid binding
AAEL013339	Lethal (2) essential for life protein l2efl	4.573305	Multicellular organism development
AAEL014937	Hypothetical protein	1.519918	Not annotated
AAEL012013	Hypothetical protein	4.487515	Chitin metabolic process
AAEL001785	Origin recognition complex subunit	1.864977	Origin recognition complex
AAEL015047	Hypothetical protein	4.127470	Not annotated
AAEL018047	Not annotated	−2.099707	Not annotated
AAEL004231	M12 mutant protein precursor 2C putative	−2.142678	Cell motility

**Table 6:** Components of Wnt pathway and Notch pathway/neuronal development genes that are differentially expressed in two field populations compared to ROCK population four days after CHIKV infection.

Gene ID	Fold Change (log2)	Description	GO ID	GO Function Description
KW				
Wnt signaling pathway				
AAEL008847	2.600	wingless	GO:0016055	Wnt signaling pathway
AAEL004932	2.320	tyrosine-protein kinase	GO:0008543	fibroblast growth factor receptor signaling pathway
AAEL001235	−1.628	palmitoyl-protein thioesterase	GO:0008474	palmitoyl-(protein) hydrolase activity
AAEL007828	−1.595	palmitoyl-protein thioesterase	GO:0008474	palmitoyl-(protein) hydrolase activity
AAEL015038	2.124	palmitoyl-protein thioesterase	GO:0008474	palmitoyl-(protein) hydrolase activity
AAEL011695	3.224	conserved hypothetical protein	GO:0035023	regulation of Rho protein signal transduction
AAEL001530	−1.835	hypothetical protein	GO:0035023	regulation of Rho protein signal transduction
AAEL000734	2.840	hydroxysteroid dehydrogenase	GO:0035023	regulation of Rho protein signal transduction
AAEL009870	5.880	low-density lipoprotein receptor (ldl)	GO:0019013	viral nucleocapsid
AAEL000324	1.870	tyrosine-protein kinase drl, Wnt-activated receptor	GO:0004713	protein tyrosine kinase activity
AAEL011773	2.199	Calreticulin, Wnt signaling regulator	GO:0005783	endoplasmic reticulum; coreceptor for wnt protein
AAEL001074	−2.103	cadherin	GO:0007156	homophilic cell adhesion via plasma membrane adhesion molecules
AAEL006540	7.155	rab	GO:0007264	small GTPase mediated signal transduction
Notch signaling pathway/neuronal function and development				
AAEL017503	2.952	NA	GO:0007219	Notch signaling pathway
AAEL004219	2.340	rap GTPase-activating protein	GO:0051056	regulation of small GTPase mediated signal transduction
AAEL003586	2.464	neuronal cell adhesion molecule	GO:0005515	protein binding
AAEL000243	2.347	leucine-rich transmembrane protein	GO:0005515	protein binding
AAEL002307	−4.061	leucine-rich transmembrane protein	GO:0005515	protein binding
AAEL003720	−2.366	leucine-rich transmembrane protein	GO:0005515	protein binding
AAEL007231	2.581	leucine-rich immune protein (Coil-less)	GO:0005515	protein binding
AAEL001106	2.242	von Hippel-Lindau disease tumor suppressor C putative	GO:0042073	intraciliary transport
AAEL012001	−2.588	Galectin, JNK regulator	GO:0030246	carbohydrate binding
Notch signaling pathway/neuronal function and development				
AAEL005507	3.411	Inhibitory pou neural development	GO:0006351	transcription, DNA-templated
AAEL003586	2.025	neuronal cell adhesion molecule	GO:0005515	protein binding
AAEL003720	−1.878	leucine-rich transmembrane protein	GO:0005515	protein binding

**Table 7:** Validation of expression of transcripts in field population compared to a lab population, ROCK, by qRT-PCR (*: *p* < 0.05, **: *p* < 0.005).

Accession Number	AAEL000912	AAEL008847	AAEL015038	AAEL007220
qRT-PCR_Field/Rock	12.8162 **	14.773 **	2.42006 *	13.9022 *
RNAseq_Field/Rock	1.749	2.6	2.124	3.183

## Supplementary Materials

The following are available online at https://www.mdpi.com/1999-4915/12/8/823/s1. Table S1: Selected genes and primer sequences for RNAseq validation; Table S2: List of differentially expressed genes between Rockefeller and Vero Beach populations related to CHIKV infection; Table S3: List of the overlapped genes in KW and Vero populations compared to ROCK.
