# Supplementary material for: Profiling Transcripts of Vector Competence between Two Different Aedes aegypti Populations in Florida
Source: Viruses. 2020 Jul 29;12(8):823. doi: 10.3390/v12080823 (PMC7472143; doi:10.3390/v12080823)
Supplement: Supplementary file 1 [file viruses-12-00823-s001.zip › viruses-831544-for conversion-suppl/supplemental tables/Table S1.pdf]

Table S1. Selected genes and primer sequences for RNAseq validation.

**Gene ID**	**Primer Sequence**	**Gene Annotation**
AAEL008847 Forward	GTT CGG AGG ACG TCA ACT AC	Wingless
AAEL008847 Reverse	CGA GCT TCA TTG TG	
AAEL015038 Forward	ACT CGA TCG GTT GCA CAA T	Palmitoyl-protein thioesterase
AAEL015038 Reverse	CGA ACG AAC GGT GAA TGA AAC	
AAEL007220 Forward	ATC GAA CAG TGG CCC AAT AC	Conserved hypothetical protein
AAEL007220 Reverse	ATG GTC GAA TGA CAG CTA TGG	
AAEL000912 Forward	GAG TTC GAT CGC TGC CTA TT	Conserved hypothetical protein
AAEL000912 Reverse	GGA ACA CTA CAT GGA CTT CTT CT	
